# Safety of CoronaVac and ChAdOx1 vaccines against SARS-CoV-2 in patients with rheumatoid arthritis: data from the Brazilian multicentric study safer

**DOI:** 10.1186/s42358-024-00397-5

**Published:** 2024-08-12

**Authors:** Vitor Alves Cruz, Camila Guimarães, Jozelia Rêgo, Ketty Lysie Libardi Lira Machado, Samira Tatiyama Miyamoto, Ana Paula Neves Burian, Laiza Hombre Dias, Flavia Zon Pretti, Danielle Cristina Filgueira Alves Batista, José Geraldo Mill, Yasmin Gurtler Pinheiro de Oliveira, Carolina Strauss Estevez Gadelha, Maria da Penha Gomes Gouveia, Anna Carolina Simões Moulin, Bárbara Oliveira Souza, Laura Gonçalves Rodrigues Aguiar, Gabriel Smith Sobral Vieira, Luiza Lorenzoni Grillo, Marina Deorce de Lima, Laís Pizzol Pasti, Heitor Filipe Surlo, Filipe Faé, Isac Ribeiro Moulaz, Mariana de Oliveira Macabú, Priscila Dias Cardoso Ribeiro, Vanessa de Oliveira Magalhães, Mariana Freitas de Aguiar, Erika Biegelmeyer, Flávia Maria Matos Melo Campos Peixoto, Cristiane Kayser, Alexandre Wagner Silva de Souza, Charlles Heldan de Moura Castro, Sandra Lúcia Euzébio Ribeiro, Camila Maria Paiva França Telles, Juliana Bühring, Raquel Lima de Lima, Sérgio Henrique Oliveira Dos Santos, Samuel Elias Basualto Dias, Natália Seixas de Melo, Rosely Holanda da Silva Sanches, Antonio Luiz Boechat, Natália Sarzi Sartori, Vanessa Hax, Lucas Denardi Dória, Rodrigo Poubel Vieira de Rezende, Katia Lino Baptista, Natália Rodrigues Querido Fortes, Ana Karla Guedes de Melo, Tâmara Santos Melo, Rejane Maria Rodrigues de Abreu Vieira, Adah Sophia Rodrigues Vieira, Adriana maria kakehasi, Anna Carolina Faria Moreira Gomes Tavares, Aline Teixeira de Landa, Pollyana Vitoria Thomaz da Costa, Valderilio Feijó Azevedo, Olindo Assis Martins-Filho, Vanessa Peruhype-Magalhães, Marcelo de Medeiros Pinheiro, Odirlei André Monticielo, Edgard Torres Dos Reis-neto, Gilda Aparecida Ferreira, Viviane Angelina de Souza, Andréa Teixeira-Carvalho, Ricardo Machado Xavier, Emilia Inoue Sato, Valeria Valim, Gecilmara Salviato Pileggi, Nilzio Antonio da Silva

**Affiliations:** 1 Universidade Federal de Goiás, Goiania, Brazil; 2 Universidade Federal do Espírito Santo, Vitoria, Brazil; 3 Universidade Federal de São Paulo, Sao Paulo, Brazil; 4 Universidade Federal do Amazonas, Manaus, Brazil; 5 Universidade Federal do Rio Grande do Sul, Porto Alegre, Brazil; 6 Universidade Federal Fluminense, Niteroi, Brazil; 7 Universidade Federal da Paraíba, Joao Pessoa, Brazil; 8 Hospital Geral de Fortaleza, Fortaleza, Brazil; 9 Universidade de Fortaleza, Fortaleza, Brazil; 10 Universidade Federal de Minas Gerais, Belo Horizonte, Brazil; 11 Universidade Federal de Juiz de Fora, Juiz de Fora, Brazil; 12 Universidade Federal do Paraná, Curitiba, Brazil; 13 Instituto Renè Rachou, Belo Horizonte, Brazil

**Keywords:** Vaccine, COVID-19, Rheumatoid arthritis

## Abstract

**Background:**

Patients with immune-mediated rheumatic diseases (IMRDs) have been prioritized for COVID-19 vaccination to mitigate the infection severity risks. Patients with rheumatoid arthritis (RA) are at a high risk of severe COVID-19 outcomes, especially those under immunosuppression or with associated comorbidities. However, few studies have assessed the safety of the COVID-19 vaccine in patients with RA.

**Objective:**

To evaluate the safety of vaccines against SARS-CoV-2 in patients with RA.

**Methods:**

This data are from the study “Safety and Efficacy on COVID-19 Vaccine in Rheumatic Diseases,” a Brazilian multicentric prospective phase IV study to evaluate COVID-19 vaccine in IMRDs in Brazil. Adverse events (AEs) in patients with RA of all centers were assessed after two doses of ChAdOx1 (Oxford/AstraZeneca) or CoronaVac (Sinovac/Butantan). Stratification of postvaccination AEs was performed using a diary, filled out daily and returned at the end of 28 days for each dose.

**Results:**

A total of 188 patients with RA were include, 90% female. CoronaVac was used in 109 patients and ChAdOx1 in 79. Only mild AEs were observed, mainly after the first dose. The most common AEs after the first dose were pain at the injection (46,7%), headache (39,4%), arthralgia (39,4%), myalgia (30,5%) and fatigue (26,6%), and ChAdOx1 had a higher frequency of pain at the injection (66% vs 32 %, *p* < 0.001) arthralgia (62% vs 22%, *p* < 0.001) and myalgia (45% vs 20%, *p* < 0.001) compared to CoronaVac. The more common AEs after the second dose were pain at the injection (37%), arthralgia (31%), myalgia (23%), headache (21%) and fatigue (18%). Arthralgia (41,4% vs 25%, *p* = 0.02) and pain at injection (51,4% vs 27%, *p* = 0.001) were more common with ChAdOx1. No serious AEs were related. With Regard to RA activity level, no significant difference was observed between the three time periods for both COVID-19 vaccines.

**Conclusion:**

In the comparison between the two immunizers in patients with RA, local reactions and musculoskeletal symptoms were more frequent with ChAdOx1 than with CoronaVac, especially after the first dose. In summary, the AE occurred mainly after the first dose, and were mild, like previous data from others immunizing agents in patients with rheumatoid arthritis. Vaccination did not worsen the degree of disease activity.

## Introduction

The SARS-CoV-2 (severe acute respiratory syndrome coronavirus 2) has infected millions of people around the world, with approximately 769 million cases and 6.9 million deaths accumulated up to August 2023. Brazil is amongst the most affected countries, with 37.7 million confirmed cases and approximately 705,000 SARS-CoV2 related deaths. There have been at least three main waves of the disease, triggered respectively by the original Wuhan strain and its gamma and omicron variants. The gamma was considered the most contagious and lethal one, with a higher risk of hospitalization of healthy young adults. In this scenario, vaccines were eagerly awaited to reduce the mortality and morbidity of COVID-19 (Coronavirus disease 2019) [1].

Immunocompromised patients, such as those who take immunosuppressive drugs, transplant recipients or those with immune-mediated chronic inflammatory diseases, are considered to be at greater risk for severe manifestations of COVID-19 [2]. However, data from registry studies, including the Brazilian ReumaCov [3], indicate that not every immunosuppressed patient is at increased risk of severe disease. Certain immunobiological therapies, especially TNF (tumor necrosis factor alpha) inhibitors, were correlated with lower odds of hospitalization in cohorts of patients with rheumatic diseases, chronic inflammatory bowel diseases and psoriasis [4]. The most important factors associated with an increased risk of hospitalization and death in patients with immune-mediated rheumatic diseases were advanced age, high activity of the underlying disease, use of glucocorticoids (in doses equivalent to prednisolone ≥ 10 mg) and B-cell depleting drugs such as rituximab [3]. On the other hand, drugs used in the management of IMRDs (immune-mediated rheumatic diseases) have been utilized as potential treatments for COVID-19, especially in mitigating the so-called cytokine storm, some of which, such as Baricitinib and Tocilizumab, have shown considerable benefits [5, 6].

Vaccination against SARS-CoV-2 is already a consolidated reality for the most vulnerable groups, including patients receiving immunosuppressants. It is known, however, that older patients using corticosteroids and with high activity of the underlying disease respond less adequately to vaccinations [7–10]. The data we currently have available still leaves doubts regarding vaccination against SARS-CoV-2 in patients with rheumatoid arthritis (RA), such as the its impact on the degree of disease activity and possible differences in relation to the safety of different immunizers.

Vaccination uptake of patients with RA, even in academic centers, has proved to be suboptimal [11]. It is recommended to vaccinate patients preferably before initiation of immunosuppression. Live attenuated vaccines should be avoided when using disease-modifying antirheumatic drugs (DMARDs). Non-live vaccines are safe during immunosuppression, despite the potential impairment of immunogenicity, especially when rituximab, abatacept, methotrexate, and glucocorticoids are used, with a similar safety profile to the general population [12–15].

In the present study, we aimed to prospectively evaluate the safety of ChAdOx1 and CoronaVac, the two immunizers available in Brazil at the beginning of vaccination of patients with comorbidities, in a cohort of patients with RA, as well as to compare the impact of these COVID-19 vaccines on RA disease activity.

## Methodology

This study was a subanalysis of the SAFER project (Study of Safety, Effectiveness and Duration of Immunity after Vaccination against SARS-CoV-2 in Patients with Immune-Mediated Chronic Inflammatory Diseases), an observational longitudinal multicenter study sponsored by the Brazilian Society of Rheumatology (BSR) and the Department of Science an Technology (DECIT) of the Ministry of Health of Brazil. According to the study protocol, patients with RA vaccinated with either the inactivated adsorbed vaccine registered by the Butantã Institute (CoronaVac) or the non-replicating viral vector vaccine registered by Bio-Manguinhos/Fiocruz (ChAdOx1), were assessed at prespecified time points. During these encounters, they collected blood samples for analyses, underwent physical examination, and also responded questions from a standardized questionnaire on new symptoms suggestive of COVID. Patients received immunization at vaccination centers in the cities of origin, based on medical advice on the best time for vaccination, and were followed up from the day of the first dose. The platform Redcap was used to store the SAFER dataset.

Between June, 2021 and September, 2021, patients classified as RA according to the 2010 ACR/EULAR (American College of Rheumatology/European Alliance of Associations for Rheumatology) classification criteria, on stable treatments for at least three months and aged 18 years or older were included in the study. The participants were recruited from 9 tertiary hospitals across all Brazilian macroregions (convenience sample). The exclusion criteria were as follows: Individuals with a history of serious adverse events related to any type of vaccine, pregnant women, minors, primary immunodeficiencies, HIV (human immunodeficiency virus), neoplasms and thymus diseases. Vaccination was postponed in those with a history of using any blood product, pulse therapy with cyclophosphamide or plasmapheresis in the previous 30 days. It was also delayed for at least four weeks in cases of suspicion or confirmation of covid. As for the use of rituximab, inclusion was allowed no earlier than six months from the last infusion.

The follow-up of the patients was carried out with visits to assess the safety of the immunizer and analysis of disease activity, before immunization and four weeks after each of the two doses. According to the National Immunization Plan put forward by the Ministry of Health of Brazil and the recommendations of the BSR [16], in effect at the time of the study, the between-dose intervals were 28 days for CoronaVac and 90 days for ChAdOx1 [17].

The monitoring of Short-term adverse events was carried out by filling in a diary to record possible symptoms, updated daily for 4 weeks, and then returned to the center in the next scheduled visit, happened one month after immunization. RA activity was scored with the Clinical Disease Activity Index (CDAI). We compared the CDAI values obtained in the three months before COVID-19 vaccination, collected from the patients chart, to that at four weeks following each immunization, performed by the investigators.

Data were tabulated and analyzed using Stata software version 14 (StataCorp, USA). Proportions between groups were compared using the chi-square or Fisher’s exact tests, as appropriate, for categorical variables. Mean and standard deviation, as well as median and interquartile range were calculated for continuous variables and analyzed using ANOVA and Kruskal-Wallis, respectively. Disease activity before and after vaccination was assessed using the McNemar test. All analyses were performed using the statistical package Stata version 17. For all tests, a statistical significance level of less than 5% and a confidence interval of 95% were used.

## Results

This study corresponds to one of the arms of the SAFER Project, with RA patients vaccinated according to medical advice, carried out in Brazil from June to September 2021 in which individuals with IMRDs were followed up before and after the first two doses of the basic vaccination schedule. A total of 199 patients with RA were eligible for the study and, of these, 11 were excluded because they did not receive the CoronaVac or ChAdOx1 vaccines, resulting in 188 participants.

Of these 188 patients, the majority of them were female (90.4%), brown (51.6%), and middle-aged (mean 45.9 years). Arterial hypertension and obesity were the most frequent comorbidities (26.6% and 13.3%, respectively) (Table 1). The participating centers that first obtained ethics committee approval accounted for approximately approximately 75% of the patients (Table 2).

**Table 1:** Clinical-epidemiological variables of SAFER project patients with rheumatoid arthritis according to types of immunizers

Variables	Total	CoronaVac	ChAdOx1	*P*
	*N* = 188 (100%)	*N* = 109 (58%)	*N* = 79 (42%)	
Sex				0.084
Male	18 (9.5%)	14 (12.8%)	4 (5%)	
Female	170 (90.4%)	95 (87.1%)	75 (94.9%)	
Age (Years), Average (SD)	45.9 (12.4)	44.3 (13.1)	48.0 (11.2)	0.047
Race/ethnicity				0.66
White	66 (35.1%)	36 (33.0%)	30 (37.9%)	
Black	21 (11.1%)	11 (10%)	10 (12.6%)	
Brown	97 (51.6%)	60 (55%)	37 (46.8%)	
Indigenous	1 (0.5%)	0 (0%)	1 (1.2%)	
Asian	3 (1.6%)	2 (1.8%)	1 (1.2%)	
BMI (Kg/m^2^), Average (SD)	26.7 (5.5)	27.1 (5.40)	26.2 (5.8)	0.28
No comorbidities	79 (42%)	48 (44%)	31 (39.2%)	0.51
Cardiac condition	3 (1.6%)	3 (2.7%)	0 (0%)	0.27
Diabetes	12 (6.3%)	6 (5.5%)	6 (7.5%)	0.56
Lung disease	6 (3.1%)	5 (4.5%)	1 (1.2%)	0.40
Kidney disease	0 (0%)	0 (0%)	0 (0%)	
Hypertension	50 (26.6%)	24 (22%)	26 (32.9%)	0.095
Obesity	25 (13.3%)	12 (11%)	13 (16.4%)	0.28
Methotrexate	106 (56.3%)	66 (60%)	40 (50.6%)	0.18
Leflunomide	46 (24.4%)	28 (25.6%)	18 (22.7%)	0.65
Hydroxychloroquine	24 (12.7%)	13 (11.9%)	11(13.9%)	0.69
Anti-TNF	49 (26%)	24 (22%)	25 (31.6%)	0.14
Tocilizumab	21 (11.1%)	10 (9.1%)	11 (13.9%)	0.31
Abatacept	12 (6.3%)	9 (8.2%)	3 (3.8%)	0.25

*SD* standard deviation, *BMI* body mass index

**Table 2:** Distribution of SAFER project patients with rheumatoid arthritis, according to state of origin

Variables	Total
	*N* = 188 (100%)
FU	
Amazonas (AM)	30 (15.9%)
Ceará (CE)	10 (5.3%)
Espírito Santo (ES)	59 (31.3%)
Goiás (GO)	29 (15.4%)
Paraíba (PB)	8 (4.2%)
Paraná (PR)	1 (0.5%)
Rio de Janeiro (RJ)	11 (5.8%)
Rio Grande do Sul (RS)	9 (4.7%)
São Paulo (SP)	31 (16.4%)

*FU* federative unit

Regarding the treatment of RA, 51% of patients used a regimen with only conventional synthetic drugs, while 47% used an immunobiological and 2% a target-specific synthetic drug. Corticosteroids were used in 26%, with a mean dose of 7.5 mg of prednisone. The most used drugs were methotrexate (56%), anti-TNFs (26%) and leflunomide (24%). There was no statistical difference in relation to the treatment used in patients vaccinated with ChAdOx1 and CoronaVac.

Comparison of clinical and epidemiological variables between the two types of immunizers that showed a statistical difference only in the age variable (Table 1).

Local reactions, especially injection-site pain, were the most common adverse event up to 28 days after the first vaccination. Musculoskeletal symptoms and headache were also frequently reported. Symptoms ranged from two to four days. Injection site reactions, fever, headache, dizziness, fatigue, and musculoskeletal symptoms were significantly more prevalent with ChAdOx1 than with CoronaVac. Eight patients did not complete adverse event diaries and were excluded from this subanalysis (Table 3).

**Table 3:** Adverse events after the first vaccine dose according to the type of immunization in SAFER project patients with rheumatoid arthritis

Adverse events 28 days after 1st dose	Total	CoronaVac	ChAdOx1	*P*
	*N* = 180 (100%)	*N* = 105 (58%)	*N* = 75 (42%)	
Erythema around the injection site	20 (11.1%)	3 (2.8%)	17 (22.6%)	<0.001
Bruising or redness around the injection site	20 (11.1%)	4 (3.8%)	16 (21.3%)	<0.001
Swelling around the injection site	35 (19.4%)	9 (8.5%)	26 (34.6%)	<0.001
Tightening of the skin around the injection site	33 (18.3%)	7 (6.6%)	26 (34.6%)	<0.001
Soreness in the area that received the injection	85 (47.2%)	34 (32.3%)	51 (68%)	<0.001
Nausea and/or vomiting	31 (17.2%)	14 (13.3%)	17 (22.6%)	0.10
Fatigue	48 (26.6%)	20 (19%)	28 (37.3%)	0.006
Headache	71 (39.4%)	32 (30.4%)	39 (52%)	0.004
Muscle pain	55 (30.5%)	21 (20%)	34 (45.3%)	<0.001
Joint pain	71 (39.4%)	24 (22.8%)	47 (62.6%)	<0.001
Fever	31 (17.2%)	10 (9.5%)	21 (28%)	0.001
Dizziness	37 (20.5%)	13 (12.3%)	24 (32%)	0.001

After the second dose, the main self-reported adverse events in the short-term were local reactions. The mean duration of symptoms was two to four days. Pain, swelling, tightening of the skin at the injection site and arthralgia were more frequent with the immunizer ChAdOx1. Two patients decided not to be vaccinated and sixteen did not complete the adverse event diary, thus being excluded from this sub analysis (Table 4).

**Table 4:** Adverse events after the second vaccine dose according to the type of immunization in SAFER project patients with rheumatoid arthritis

Adverse events 28 days after 2nd dose	Total	CoronaVac	ChAdOx1	*P*
	*N* = 170 (100%)	*N* = 100 (58%)	*N* = 70 (42%)	
Erythema around the injection site	18 (10.5%)	7 (7%)	11 (15.7%)	0.069
Bruising or redness around the injection site	6 (3.5%)	1 (1%)	5 (7.1%)	0.083
Swelling around the injection site	21 (12.3%)	8 (8%)	13 (18.5%)	0.039
Tightening of the skin around the injection site	20 (11.7%)	6 (6%)	14 (20%)	0.005
Soreness in the area that received the injection	63 (37%)	27 (27%)	36 (51.4%)	0.001
Nausea and/or vomiting	18 (10.5%)	9 (9%)	9 (12.8%)	0.42
Fatigue	31 (18.2%)	14 (14%)	17 (24.2%)	0.087
Headache	37 (21.7%)	22 (22%)	15 (21.4%)	0.93
Muscle pain	39 (22.9%)	19 (19%)	20 (28.5%)	0.15
Joint pain	54 (31.7%)	25 (25%)	29 (41.4%)	0.020
Fever	18 (10.5%)	7 (7%)	11 (15.7%)	0.069
Dizziness	17 (10%)	10 (10%)	7 (10%)	1.00

CDAI score was available for 171 patients at study enrolment, 150 at four weeks following the first vaccination, and 165 at four weeks after the second vaccination. With Regard to RA activity level, no significant difference was observed between the three time periods for both COVID-19 vaccines (Table 5) or ChAdOx1 (Table 6).

**Table 5:** Degree of disease activity, measured by CDAI, after the first and second dose of Coronavac vaccines in SAFER project patients with rheumatoid arthritis

Disease activity	Inclusion	After 1st dose	*P*	After 2nd dose	*P*
	*N* = 101	CoronaVac		CoronaVac	
		*N* = 86		*N* = 96	
Remission	13 (12.8%)	13 (15.1%)	1.000	12 (12.5%)	0.796
Low activity	44 (43.5%)	36 (41.8%)	0.655	46 (47.9%)	0.578
Moderate activity	20 (19.8%)	20 (23.2%)	0.532	22 (22.9%)	0.480
High activity	24 (23.7%)	17 (19.7%)	0.197	16 (16.6%)	0.221

*P* value refers to the comparison of 4 weeks after the first dose vs. baseline and 4 weeks after second dose vs. baseline. CDAI interpretation: ≤ 2.8 = Remission; > 2.8 and ≤ 10 = Low disease activity; > 10 and ≤ 22 = Moderate disease activity; > 22: High disease activity

**Table 6:** Degree of disease activity, measured by CDAI, after the first and second doses of ChAdOx1 vaccines in SAFER project patients with rheumatoid arthritis

Disease activity	Inclusion	After 1st dose	*P*	After 2nd dose	*P*
	*N* = 70	ChAdOx1		ChAdOx1	
		*N* = 64		*N* = 69	
Remission	11 (15.7%)	7 (10.9%)	0.257	19 (27.5%)	0.090
Low activity	20 (28.5%)	19 (29.6%)	0.346	19 (27.5%)	0.835
Moderate activity	21 (30%)	19 (29.6%)	0.532	19 (27.5%)	0.695
High activity	18 (25.7%)	19 (29.6%)	0.637	12 (17.3%)	0.317

*P* value refers to the comparison of 4 weeks after the first dose vs. baseline and 4 weeks after second dose vs. baseline. CDAI interpretation: ≤ 2.8 = Remission; > 2.8 and ≤ 10 = Low disease activity; > 10 and ≤ 22 = Moderate disease activity; > 22: High disease activity

## Discussion

In this prospective Brazilian multicentric study with RA adults, we found no new safety signals with a two-dose schedule of both ChAdOx1 and CoronaVac vaccines. However, adverse events, either local or systemic, were more often reported with ChAdOx1 vaccine than with CoronaVac vaccine. Of note, none of the RA patients primed with ChAdOx1 had adverse events leading to discontinuation of vaccination. As another major finding of our study, the COVID-19 vaccines studied did not associate with worsening of RA disease activity level.

In the present study, most patients with RA were female, brown, with a mean age of 45, data similar to other previously puplished studies [18–21]. In our sample, the average age in the 45-year-old range is justified by the low inclusion of elderly people, most of whom had already been vaccinated when the project started, in May 2021, making their participation in this cohort unfeasible.

In patients with RA, comorbidities are frequent [22, 23] and increase the risk of complications and death related to COVID-19. They were also essential to establish the order of priority for vaccination. In our study, the most frequent were arterial hypertension and obesity.The high frequency of cardiovascular diseases in general, and especially of arterial hypertension, is common data in RA studies [24, 25], regardless of the population. Such finding reflects the chronic effects of pro-inflammatory cytokines and corticosteroids on the cardiovascular system, especially on the endothelium, resulting in accelerated atherosclerosis and increased peripheral vascular resistance [26], which can increase the risk of complications in COVID-19.

In the present analysis, the most frequent adverse event following immunization (AEFI) were local reactions, headache and musculoskeletal symptoms, data compatible with other studies in the population with IMRDs [27–30]. Medeiros-Ribeiro and collaborators, in their study, followed 910 individuals with IMRDs who were vaccinated with two doses of CoronaVac, including 256 patients with RA. No moderate/serious adverse events were reported. The most reported vaccine reactions were pain at the injection site (19.8%), headache (20.2%) and drowsiness (13.6%) [27].

In an Indian cohort, Cherian et al followed 724 patients with IMRDs, including 225 with RA, who used ChAdOx1 (87.1%) as the main immunizer. The main AEFI reported were pain at the injection site (24.9%), fever (18.3%), fatigue (17.9%) and headache (13.8%) [28]. In the COVAX registry, 4604 patients with IMRDs were followed, including 1686 patients with rheumatoid arthritis. The m-RNA platforms were the most used, with ChAdOx1 being used in 17% of the cases. The main adverse events reported were pain at the injection site (19%), fatigue (12%), myalgia (7%) and fever (7%) [29].

In a meta-analysis, Tang et al analyzed 4,433 patients with IMRDs vaccinated against COVID-19 with the different immunizers approved by the WHO. Local pain was the most prevalent symptom (30–55%), followed by fatigue (19–28%). Only two serious adverse events were reported [30].

In line with previously published data, this cohort reinforces the safety of ChAdOx1 and CoronaVac vaccines against SARS-CoV-2 in the RA population with only mild adverse events occurring. A variable frequency of AEFIs is observed among the different studies, which can be justified by the analysis encompassing, simultaneously, different inflammatory diseases and distinct immunizers. More studies are needed to clarify possible differences in the frequency of adverse events following immunization among different immune-mediated rheumatic diseases.

In the monitoring of our group, AEFI were more frequent after the first dose of immunizers, corroborating data previously published in studies with the general population and in cohorts including patients with IMRDs [27, 30, 31]. In the meta-analysis by Tang et al, AEFI were more common after the first dose of the non-replicating viral vector platform, especially pain at the site of injection, myalgia and fever [30]. In the study by Gopaul et al, adverse effects were milder and shorter in duration after the second dose of ChAdOx1 [31]. Medeiros et al observed a lower frequency of pain at the injection site, fever, myalgia and arthralgia after the second dose of CoronoVac [27]. Such findings may be related to the mechanism of action and immunogenicity of each platform, considering that published data with m-RNA vaccines show the exact opposite, with more intense reactions after the second dose [32].

AEFIs were more frequent with ChAdOx1 in comparison to CoronaVac, similar to data observed in the analysis of population studies with the two immunizers in healthy individuals. In the study by Riad et al, with the immunizer Coronavac, the frequency of local AEFI was 41% [33]. In a multicenter study, in which the immunizer was ChAdOx1, Falsey et al found 74% of adverse events following immunization [34]. In the study by Esquivel et al, 60% of patients who received ChAdOx1 immunization and 36% of patients who received CoronaVac immunization had pain at the injection site [35]. These results support the hypothesis that the greater immunogenicity of the non-replicating viral vector platform, compared to that of inactivated virus, would be related to a higher frequency of AEFI [36].

In line with our data, prior studies in RA and other IMRDs populations have not shown significant flare-up of disease activity following immunization with different types of COVID-19 vaccines. In the COVAX registry, 5% of patients with inflammatory arthropathy had reactivation of the underlying disease after immunization [29]. Li et al analyzed 5,493 patients with RA, of which 671 were vaccinated with CoronaVac, and found no association between the immunizer and the worsening of disease activity [37]. Bixio et al observed worsening of pain and joint swelling in 7.8% of patients after vaccination [38]. In analyzes involving other immune-mediated rheumatic diseases, no worsening of disease activity was observed after vaccination against SARS-CoV-2 [39, 40]. Connolly et al. described that vaccine platforms with mRNA mechanism of action did not influence the evolution of diffuse connective tissue diseases [39]. Pinte et al observed a similar incidence of IMRD reactivation in vaccinated and unvaccinated groups (6% versus 8%, *p* = 0.302). Furthermore, the two groups did not show any significant difference, not even in the duration of symptoms [40].

RA patients may hesitate to be vaccinated mainly due to safety concerns, especially the risk of relapse after vaccination [41]. It is discussed whether SARS-CoV-2 could trigger cross-reactivity through molecular mimicry, leading to autoimmunity, and whether immunizers containing the antigens of this agent could induce similar effects. Studies with chronic immune-mediated inflammatory diseases, in general, have refuted this hypothesis, reaffirming that the immunization of these patients is safe. Such findings are essential to overcome vaccine hesitancy and increase protection rates in this group [39, 40].

Over the last few years, several immunizers have been studied without any significant risk of worsening symptoms related to IMRDs being observed. In distinct scenarios where reactivation is observed, it is generally of mild intensity, short duration and does not require an increase in the degree of immunosuppression [42–44]. Systematic vaccination of patients with RA is recommended by the BSR and reaffirmed in international guidelines such as the EULAR [12, 45].

Although immunosuppressed patients were excluded from phase 3 clinical trials of the efficacy and safety of immunizers against SARS-CoV-2, their vaccination was considered a priority due to the increased risk of hospitalization and death from COVID-19. In this study, both immunizers had a good safety profile.

Our cohort has limitations regarding its sample, with low representation of elderly people, non-standardization of baseline assessment of disease activity and the observational design, making it impossible to control the formation of the groups studied. Also noteworthy is the complex scenario where the vaccination schedule for patients with comorbidities was not standardized in all regions of Brazil, in addition to local differences in the speed of processing documents in the respective Ethics Committees, limiting the inclusion of patients in some centers. Although there is still no universally accepted definition of flare by CDAI, another limitation was the analysis model of the disease activity using only distribution in range, since the present study did not account for other definitions, such as change in categories (ex: remission/low disease activity to moderate/high disease activity) or by variation of CDAI value, based on the increase by 4.5, as proposed by Konzett et al. [46].

Our data cannot be generalized due to convenience sampling and the relatively small number of patients, however, the baseline demographic and clinical characteristics of our population were similar to that in the literature.

## Conclusions

ChAdOx1 and CoronaVac vaccines are safe in RA patients. The frequency of local adverse effects, particularly pain at the injection site, is high. AEFIs are more frequent with ChAdOx1, especially after the first dose. The use of the immunizers Coronavac and ChAdOx1 does not change the degree of inflammatory activity of the disease.

## Data Availability

Not applicable.
